# Single inferior parathyroid autotransplantation during total thyroidectomy with bilateral central lymph node dissection for papillary thyroid carcinoma: a retrospective cohort study

**DOI:** 10.1186/s12957-023-02886-1

**Published:** 2023-03-23

**Authors:** Zhizhong Dong, Wen Liu, Ying Peng, Xiangxiang Zhan, Yanjun Su, Chang Diao, Ruochuan Cheng

**Affiliations:** Department of Thyroid Surgery, Clinical Research Center for Thyroid Diseases of Yunnan Province, The First Affiliated Hospital of Kunming Medical University, Kunming, China

**Keywords:** Papillary thyroid carcinoma, Parathyroid autotransplantation, Hypoparathyroidism, Central lymph node dissection, Quality of life

## Abstract

**Background:**

Management of the inferior parathyroid gland using total thyroidectomy (TT) with central lymph node dissection (CLND) is still controversial. Therefore, we evaluated the safety and effectiveness of single inferior parathyroid autotransplantation.

**Methods:**

The clinical data of patients with papillary thyroid carcinoma (PTC) who underwent TT with bilateral CLND from January 2018 to December 2020 were collected. Quality of life (QoL) was assessed using the Chinese version of the EORTC QLQ-C30 and THYCA-QOL. The patients were divided into an autotransplantation group and a preservation group according to whether a single inferior parathyroid gland was transplanted. The incidence of permanent hypoparathyroidism, the number of resected central lymph nodes (CLNs), the rate of recurrence reoperation, the rate of radioactive iodine (RAI) treatment, and the QoL score were compared between the two groups.

**Results:**

A total of 296 patients were included in the study; there were 99 patients in the autotransplantation group and 197 in the preservation group. The incidence of permanent hypoparathyroidism was 3.0% (3/99) and 4.6% (9/197) in the autotransplantation and preservation groups, respectively (*P* = 0.532). The median number of resected CLNs was 12 (8–17) and 10 (6–14) in the autotransplantation and preservation groups, respectively (*P* = 0.015). No reoperations were performed for patients with CLN recurrence, and the rates of lateral lymph node (LLN) recurrence reoperation were 2.0% (2/99) and 3.6% (7/197) in the autotransplantation and preservation groups, respectively (*P* = 0.473). The RAI treatment rates were 12.1% (12/99) and 22.3% (44/197) in the autotransplantation and preservation groups, respectively (*P* = 0.034). A total of 276 questionnaires were recovered, including 84 in the autotransplantation group and 192 in the preservation group. The QoL of the two groups of patients is similar (*P* > 0.05).

**Conclusion:**

Single inferior parathyroid autotransplantation during thyroidectomy can be used to prevent permanent hypoparathyroidism and can enable more extensive CLND.

## Introduction

In recent years, the incidence of papillary thyroid carcinoma (PTC) has increased substantially, and it has become the most common malignant tumor of the endocrine system in the world [1–3]. Hypoparathyroidism is one of the most common and serious complications after thyroid surgery. Studies have shown that the incidence of permanent hypoparathyroidism after thyroid surgery is approximately 0–20.2% [4, 5]. The main causes of hypoparathyroidism are intraoperative parathyroid injury, devascularization, and unintentional resection [6, 7]. Ideally, preserving each parathyroid gland in situ during surgery is optimal; however, this is challenging even for high-volume surgeons [8]. Due to the lack of ideal methods to judge the survival of the parathyroid gland during surgery, the selection of parathyroid preservation in situ or autotransplantation mainly depends on the operation and experience of the surgeon. The superior parathyroid gland is easily to preserve in situ, but the inferior parathyroid gland is difficult to be preserved in situ during central lymph node dissection (CLND) because of its variable location and single blood supply. Expansion of the CLND scope increases the possibility of injury to the parathyroid gland and its blood supply [9]. Therefore, protection of parathyroid function while ensuring complete CLND, reducing tumor recurrence, and improving the quality of life (QoL) of patients have been problematic in the field of thyroid surgery.

At present, the management of parathyroid glands, especially inferior parathyroid glands, during thyroid surgery is controversial. In this study, PTC patients who underwent total thyroidectomy (TT) and bilateral CLND were included. The effect of single inferior parathyroid autotransplantation on permanent hypoparathyroidism, the number of resected central lymph nodes (CLNs), recurrence reoperation, radioactive iodine (RAI) treatment, and postoperative QoL were retrospectively analyzed to provide a reference for the safety and effectiveness of parathyroid autotransplantation during thyroid surgery.

## Materials and methods

### Patients

The clinical data of PTC patients who underwent TT with bilateral CLND for the first time in the Department of Thyroid Surgery of the First Affiliated Hospital of Kunming Medical University from January 2018 to December 2020 were retrospectively analyzed. Patients who had single inferior parathyroid autotransplantation and patients who had no parathyroid autotransplantation were included. All patients did not take calcium supplements and vitamin D supplements for a long time before operation. The exclusion criteria included the following: (1) patients aged < 18 years, (2) patients whose preoperative serum parathyroid hormone (PTH) and calcium levels were abnormal, (3) the presence of parathyroid tissue on the thyroid gland during histopathological examination, (4) incomplete follow-up data, and (5) patients who were lost to follow-up. The patients were divided into two groups: the autotransplantation group (single inferior parathyroid autotransplantation) and the preservation group (without autotransplantation). This study was approved by the Ethics Committee of the First Affiliated Hospital of Kunming Medical University (2017 Lun Shen L No. 17).

The QoL questionnaires were distributed to the enrolled patients through WeChat, including the Chinese versions of the European Organization for Research and Treatment of Cancer Quality of Life Questionnaire-C30 (EORTC QLQ-C30) and the Thyroid Cancer-specific Quality of Life (THYCA-QoL).

In the initial cohort of 316 patients, 2 patients died of causes unrelated to thyroid cancer at the last follow-up, and 18 patients were lost to follow-up and were excluded from the cohort, with a loss rate of 5.7%. A total of 296 patients were enrolled, including 99 in the autotransplantation group and 197 in the preservation group. A total of 296 questionnaires were distributed; 276 questionnaires were recovered, including 84 questionnaires from the autotransplantation group and 192 questionnaires from the preservation group, with a recovery rate of 93.2%.

### Surgical program

All surgeries were performed by experienced surgeons (>100 thyroidectomies/year) [10]. The indications for total thyroidectomy were as follows: (1) high risk radiation exposure or family history; (2) bilateral or multifocal PTC; (3) tumor T stage is T3 and T4; (4) bilateral central lymph node or lateral lymph node metastases; (5) high risk pathological subtypes; (6) unilateral PTC with contralateral benign thyroid tumor. The indications for BCLND were as follows: (1) bilateral PTC; (2) isthmus PTC; (3) tumor T stage is T3 and T4; (4) bilateral central lymph node or lateral lymph node metastases. The range of CLND is defined by the following margins: the upper margin is the hyoid edge, the lower margin is the suprasternal fossae, the posterior margin is the vertebral anterior fascia, and the external margin is the interior of the carotid sheath, including all paratracheal, pretracheal, and prelaryngeal lymph nodes and adipose tissues [11]. Lateral lymph node dissection (LLND) was performed in patients whose lateral lymph node metastasis was confirmed by preoperative fine-needle aspiration biopsy or suspected from computed tomography (CT) scan.

During thyroidectomy, the superior and inferior parathyroid glands were tried to be preserved in situ. The thymus is normally reserved unless it is invaded by tumor. When a parathyroid gland was nonviable or resected unintentionally, it is cut into pieces and autotransplanted into the sternocleidomastoid muscle after confirmation by intraoperative frozen biopsy.

### Postoperative management

During postoperative hospitalization, patients received daily intravenous calcium supplementation (calcium gluconate 10%, 60 ml). After discharge, patients were given oral calcium carbonate (600–3600 mg/day) and calcitriol (0.25 μg/day), which could be discontinued after 1 month if there was no symptomatic hypocalcemia. If the postoperative PTH is lower than the normal level for more than 6 months, the patient was diagnosed with permanent hypoparathyroidism [12]. The PTH normal range was defined as 12.00–88.00 pg/ml. TNM stage was performed according to the AJCC 8th [13], and recurrence risk stratification was performed according to the 2015 ATA guidelines [14].

#### Indications for RAI treatment [14]

RAI is not routinely recommended for patients with low risk of recurrence, RAI should be considered for patients with intermediate risk of recurrence, RAI should be routinely recommended for patients with high risk of recurrence, and whether to perform RAI according to the postoperative disease status (continuous tg > 1ng/mL in the postoperative TSH suppressed state, RAI should be considered).

### Statistical analysis

IBM SPSS version 26.0 was used for all statistical analyses. Continuous variables with a nonnormal distribution are expressed as the median (interquartile range (IQR)) and were analyzed using the Mann‒Whitney *U* test. Categorical variables were compared using chi-square tests or Fisher exact test. The QoL scores for the EORTC QLQ-C30 and THYCA-QoL scales were calculated according to the official scoring manual of EORTC [15]. *P* < 0.05 was considered to be statistically significant.

## Results

### Patient characteristics

A total of 296 patients with PTC, namely, 54 males (18.2%) and 242 females (81.8%), underwent TT with bilateral CLND. The median age was 43.0 (35.0–49.0) years. LLND was performed in 78 patients (26.4%). There was no significant difference in sex, age, BMI, preoperative PTH, and LLND between the autotransplantation group and the preservation group (*P* > 0.05). Table 1 shows the comparison of baseline characteristics between the two groups of patients.

**Table 1:** General information of the two groups

	Total (*n* = 296)	Autotransplantation group (*n* = 99)	Preservation group (*n* = 197)	*P*
Sex				0.985
Male	54 (18.2%)	18 (18.2%)	36 (18.3%)	
Female	242 (81.8%)	81 (81.8%)	161 (81.7%)	
Age (years)	43.0 (35.0–49.0)	42.0 (35.0–50.0)	43.0 (34.0–49.0)	0.743
Age				0.886
< 55	262 (88.5%)	88 (88.9%)	174 (88.3%)	
≥ 55	34 (11.5%)	11 (11.1%)	23 (11.7%)	
BMI	23.7 (21.8–26.3)	23.6 (22.0–26.0)	23.8 (21.7–26.3)	0.972
Preoperative PTH	44.9 (35.6–58.3)	45.0 (34.5–55.9)	44.6 (36.3–59.0)	0.546
LLND				0.202
Yes	78 (26.4%)	30 (30.3%)	48 (24.4%)	
No	218 (73.6%)	69 (69.7%)	149 (75.6%)	

*BMI* body mass index, *PTH* parathyroid hormone, *LLND* lateral lymph node dissection

### Postoperative pathological features

The pathological characteristics of the 296 patients are shown in Table 2. The tumor size of PTC ranged from 0.2 to 6.5 cm, and the median tumor size was 1.0 (0.6–1.5) cm. The number of resected CLNs is related to whether inferior parathyroid autotransplantation. The number of resected CLNs of the autotransplantation group was higher than that in the preservation group (IQR 12 vs. 10) (*P* = 0.015). Although the number of CLNMs was slightly higher in the autotransplantation group than in the preservation group, the difference was not statistically significant (*P* > 0.05). There was no significant differences between the two groups in tumor size, multifocality, ETE, T stage, N stage, TNM stage and initial recurrence risk stratification (*P* > 0.05).

**Table 2:** Comparison of pathological characteristics between the two groups

	Total (*n* = 296)	Autotransplantation group (*n* = 99)	Preservation group (*n* = 197)	*P*
Tumor size (cm)	1.0 (0.6–1.5)	1.0 (0.7–1.5)	1.0 (0.6–1.5)	0.364
Multifocality				0.840
Yes	192 (64.9%)	65 (65.7%)	127 (64.5%)	
No	104 (35.1%)	34 (34.3%)	70 (35.5%)	
ETE				0.451
Yes	22 (7.4%)	6 (6.1%)	16 (8.1%)	
No	274 (92.6%)	93 (93.9%)	171 (91.9%)	
Number of resected CLN	10 (7–15)	12 (8–17)	10 (6–14)	0.015
Number of CLNM	1 (0–4)	2 (0–5)	1 (0–3)	0.162
T stage				0.681
T1–2	272 (91.9%)	93 (93.9%)	179 (90.9%)	
T3–4	24 (8.1%)	6 (6.1%)	18 (9.1%)	
N stage				0.335
N0	116 (39.2%)	33(33.3%)	83 (42.1%)	
N1a	119 (40.2%)	43(43.4%)	76 (38.6%)	
N1b	61 (20.6%)	23(23.3%)	38 (19.3%)	
TNM				0.865^a^
I	277(93.6%)	94(94.9%)	183(92.9%)	
II	18(6.1%)	5(5.1%)	13(6.6%)	
III	1(0.3%)	0(0%)	1(0.5%)	
Recurrence risk				0.534
Low risk	215 (72.6%)	74 (74.7%)	141 (71.6%)	
Intermediate risk	70 (23.6%)	23 (23.2%)	47 (23.9%)	
High risk	11 (3.8%)	2 (2.1%)	9 (4.5%)	

*ETE* extrathyroidal extension, *CLN* central lymph node, *CLNM* central lymph node metastases

^a^Use Fisher exact test

### Clinical outcomes

The clinical outcomes of the two groups are shown in Table 3. The median follow-up time was 25.0 (18.0–31.0) months (range, 12–46 months). The median PTH level at more than 6 months after the operation was 31.7 (24.0–44.9) pg/ml. The PTH level at more than 6 months after the operation is similar in the autotransplantation group and the preservation group (*P* =0.739). There were no reoperations performed for patients with CLN recurrence. There was no statistical difference between the two groups in reoperation of lateral lymph node (LLN) recurrence (*P* =0.473). The incidence of permanent hypothyroidism in the autotransplantation group and the preservation group was 3.0% and 4.6%, respectively, with no significant difference (*P* = 0.532). The RAI treatment rate in the preservation group was higher than that in the autotransplantation group, and the difference was statistically significant (22.3% vs. 12.1%) (*P* < 0.05).

**Table 3:** Comparison of clinical outcomes between the two groups

	Total (*n* = 296)	Autotransplantation group (*n* = 99)	Preservation group (*n* = 197)	*P*
Follow-up time (months)	25.0 (18.0–31.0)	26 (18–33)	24 (18–30)	0.091
PTH (pg/ml)	31.7 (24.0–44.9)	31.5 (23.5–45.4)	31.7 (24.4–44.3)	0.739
Permanent hypoparathyroidism				0.532
Yes	12 (4.1%)	3 (3.0%)	9 (4.6%)	
No	284 (95.9%)	96 (97.0%)	188 (95.4%)	
Recurrence reoperation				
CLN	0 (0%)	0 (0%)	0 (0%)	NA
LLN	9 (3.0%)	2 (2.0%)	7 (3.6%)	0.473
RAI				0.034
Yes	56 (18.9%)	12 (12.1%)	44 (22.3%)	
No	240 (81.1%)	87 (87.9%)	153 (77.7%)	

*PTH* parathyroid hormone, *CLN* central lymph node, *LLN* lateral lymph node

### QoL scores of patients in the two groups

A total of 296 questionnaires were distributed; 276 questionnaires were finally recovered, including 84 questionnaires from the autotransplantation group and 192 questionnaires from the preservation group. The functioning scales and symptom scale scores of the EORTC QLQ-C30 and THYCA-QoL were similar between the two groups (*P* > 0.05). Table 4 shows the postoperative QoL scores of the two groups.

**Table 4:** Comparison of QoL scores between the two groups

	Autotransplantation group (*n* = 84)	Preservation group (*n* = 192)	*P*
THYCA-QoL symptom scales			
Neuromuscular	22.2 (5.6–27.8)	22.2 (11.1–22.2)	0.975
Voice	0.0 (0.0–16.7)	0.0 (0.0–33.3)	0.158
Concentration	16.7 (0.0–33.3)	16.7 (0.0–33.3)	0.824
Sympathetic	16.7 (0.0–33.3)	16.7 (0.0–33.3)	0.222
Throat/mouth problems	11.1 (0.0–22.2)	11.1 (0.0–22.2)	0.171
Psychological	25.0 (8.3–37.5)	25.0 (16.7–33.3)	0.853
Sensory	16.7 (0.0–33.3)	16.7 (16.7–33.3)	0.166
Problems with scar	0.0 (0.0–33.3)	0.0 (0.0–33.3)	0.258
Felt chilly	0.0 (0.0–33.3)	0.0 (0.0–33.3)	0.959
Tingling hands/feet	0.0 (0.0–33.3)	0.0 (0.0–33.3)	0.614
Gained weight	33.3 (0.0–33.3)	33.3 (0.0–33.3)	0.502
Headache	33.3 (0.0–33.3)	0.0 (0.0–33.3)	0.060
Less interest in sex	33.3 (0.0–33.3)	33.3 (0.0–33.3)	0.892
EORTC QLQ-C30 functioning scales			
Global health	66.7 (50.0–83.3)	66.7 (50.0–83.3)	0.684
Physical functioning	93.3 (86.7–100.0)	93.3 (86.7–100.0)	0.225
Role functioning	100.0 (100.0–100.0)	100.0 (100.0–100.0)	0.148
Emotional functioning	83.3 (66.7–100.0)	83.3 (66.7–91.7)	0.562
Cognitive functioning	83.3 (66.7–100.0)	83.3 (66.7–100.0)	0.638
Social functioning	100.0 (83.3–100.0)	100.0 (83.3–100.0)	0.414
EORTC QLQ-C30 symptom scales			
Pain	0.0 (0.0–16.7)	0.0 (0.0–16.7)	0.425
Fatigue	22.2 (0.0–33.3)	22.2 (11.1–33.3)	0.271
Nausea and vomiting	0.0 (0.0–0.0)	0.0 (0.0–0.0)	0.054
Dyspnea	0.0 (0.0–33.3)	0.0 (0.0–33.3)	0.807
Insomnia	16.7 (0.0–33.3)	33.3 (0.0–33.3)	0.170
Appetite loss	0.0 (0.0–0.0)	0.0 (0.0–0.0)	0.393
Constipation	0.0 (0.0–33.3)	0.0 (0.0–33.3)	0.464
Diarrhea	0.0 (0.0, 0.0)	0.0 (0.0–0.0)	0.430
Financial difficulties	0.0 (0.0–0.0)	0.0 (0.0–0.0)	0.782

Symptom scale: a lower score indicates fewer problems. Functioning scales: a higher score indicates a better QoL

## Discussion

TT with CLND is a common surgical method for the treatment of malignant thyroid tumors. However, a series of postoperative complications, such as hypoparathyroidism, recurrent laryngeal nerve injury, and tumor recurrence, seriously affect the QoL of patients. The incidence of permanent hypoparathyroidism after TT with bilateral CLND has been reported to be approximately 1.1–16.2% [16, 17]. In this study, the incidence of permanent hypoparathyroidism (4.1%) was in accordance with the reported incidence. In recent years, some researchers have suggested routine parathyroid autotransplantation during total thyroidectomy, which can reduce the incidence of permanent hypoparathyroidism [18–20]. Because the supply vessels of the inferior parathyroid gland are longer and the operation is more complicated when CLND is involved, the risk of injury to the inferior parathyroid gland is higher. Therefore, this study used the autotransplantation of a single inferior parathyroid gland as the test object and verified the effectiveness and safety of single inferior thyroid autotransplantation using various clinical outcomes.

### Permanent hypoparathyroidism

At present, it is still controversial that parathyroid autotransplantation can prevent permanent hypoparathyroidism after thyroid surgery. Zedenius et al. [21] proposed that the strategy of routine autotransplantation of at least one parathyroid gland in total thyroidectomy may reduce permanent hypoparathyroidism to zero. Routine autotransplantation of one parathyroid gland can avoid over-dissection of other parathyroid glands and shorten the operation time accordingly [22]. Ahmed et al. [19] found that autotransplantation of at least one parathyroid gland is a procedure with predictable outcome, and the risk of permanent hypoparathyroidism is the least. Studies have shown that parathyroid autotransplantation is associated with postoperative transient hypoparathyroidism, but it can effectively reduce the incidence of permanent hypoparathyroidism in the long term because the transplanted parathyroid gland can return to normal function after 3 to 14 weeks [23–25]. In this study, the long-term postoperative PTH levels of the two groups were similar. The use of parathyroid autotransplantation is not only an alternative to in situ preservation but also an important strategy to reduce the occurrence of permanent hypoparathyroidism after surgery [26]. Wei et al. [20] proposed that because the location of the inferior parathyroid gland is variable and its blood supply is easily damaged in CLND, routine autotransplantation of one inferior parathyroid gland during thyroidectomy with CLND could reduce the incidence of permanent hypoparathyroidism and CLN recurrence. In recent years, the role of intraoperative parathyroid autotransplantation in the prevention of permanent hypoparathyroidism has been questioned. Studies have shown that parathyroid autotransplantation does not prevent permanent hypoparathyroidism and increases the risk of transient and permanent hypoparathyroidism [27–29]. However, other studies have shown that parathyroid autotransplantation during thyroid surgery does not affect the incidence of permanent hypoparathyroidism [30–33]. Table 5 summarizes the basic information of the above studies. In this study, the incidence of permanent hypoparathyroidism was similar in the autotransplantation group and the preservation group (3.0% vs. 4.6%, *P* > 0.05). We consider that parathyroid autotransplantation may not increase the incidence of permanent hypoparathyroidism. Therefore, when the inferior parathyroid gland cannot be preserved in situ during thyroidectomy, timely parathyroid autotransplantation is a better choice. Although our research results recommend the use of parathyroid autotransplantation to prevent permanent hypoparathyroidism, more high-quality research is needed to provide evidence for routine parathyroid autotransplantation in light of ethical problems.

**Table 5:** Basic characteristics of the included studies

Author	Year	Study design	Follow-up time (months)	Sample size	Surgical method	Number of autotranslantation	Autotransplantation site	Autotransplantation group		Preservation group	
								Transient	Permanent	Transient	Permanent
Palazzo [26]	2005	R	6	1196	TT	1 2 3	NA	11.9% 15.1% 31.4%	0.77% 0.97% 0%	9.8%	0.98%
Ahmed [19]	2013	P	6	388	TT	NA	SCM	23.7%	0.3%	16.8%	2.9%
Wei [20]	2014	R	23-81	477	TT+BCLND	NA	SCM	26.5%	0.9%	25.0%	3.8%
Tartaglia [33]	2016	R	6	244	TT	NA	SCM	31.57%	3.5%	9%	1%
Kirdak [29]	2017	R	6	122	TT	NA	SCM	55.7%	11.5%	26.2%	3.3%
Su [32]	2018	R	6	702	TT	NA	SCM	43.9%	1.0%	29.0%	0.7%
Qiu [31]	2021	R	24	2477	TT	1 2	SCM	1.5% 1.0%	0.7% 0.4%	2.2%	1.7%

*R* Retrospective, *P* Prospective, *TT* Total thyroidectomy, *BCLND* Bilateral central lymph node dissection, *NA* Not acknowledge, *SCM* Sternocleidomastoid muscle

### CLND, RAI, and local recurrence

In this study, we found that the number of resected CLNs in the autotransplantation group was significantly higher than that in the preservation group (IQR 12 vs. 10, *P* = 0.015). Although the number of CLNMs in the autotransplantation group was slightly higher than that in the preservation group, there was no significant difference. This may mean that intraoperative inferior parathyroid gland autotransplantation can enable comprehensive CLND. Studies have shown that a higher number of resected CLNs at the time of primary surgery in PTC are associated with a lower rate of recurrence [34, 35]. Yu et al. [35] found that the number of resected central lymph nodes < 11 is an independent risk factor for recurrence after initial surgery (HR=4.274, *P*＜0.001). It is difficult to distinguish the inferior parathyroid gland from enlarged lymph nodes. To avoid damage to the parathyroid gland, “berry picking” CNLD may be performed, in which a complete nodal group within the compartment is not removed; however, this may be the most important reason for CNL recurrence. In our study, no CLN recurrence was observed in the two groups, and only 9 patients had LLN recurrence reoperation, with no significant difference (*P* > 0.05). Therefore, we cannot evaluate the effect of autotransplantation of an inferior parathyroid gland on the recurrence rate of CLN. We will expand the sample size and further investigate this issue after long-term follow-up in subsequent studies.

Heaton et al. [36] showed that a higher number of resected CLNs during primary PTC surgery were associated with lower recurrence rates and RAI treatment rates. Sung et al. [37] found that a larger number of resected CLNs were associated with a lower thyroglobulin (Tg) level before and after RAI treatment, and thorough CLND could improve the long-term recurrence-free survival rate. In this study, the number of resected CLNs in the autotransplantation group was much higher than that in the preservation group. In accordance with the recommendations of RAI treatment in the 2015 American Thyroid Association (ATA) guidelines [14], the preservation group has a higher proportion of RAI treatment than the autotransplantation group (12.1% vs. 22.3%, *P* < 0.05). However, in this study, the proportion of patients with intermediate and high risk of recurrence after surgery in the two groups is similar. This may be related to higher Tg levels and a lower excellent response rate in dynamic recurrence risk assessment in the preservation group. Research shows that the risk of disease recurrence increases with the increase of postoperative Tg [38, 39]. Obviously, postoperative Tg value is an important prognostic factor to guide clinical decision-making that can be used to guide clinical management. At present, we still lack complete continuous Tg value after operation, so we can not make a good prediction.

### QoL

In a questionnaire survey on the QoL of 252 patients with permanent hypoparathyroidism, nearly two-thirds of the patients believed that their hypocalcemia symptoms interfered with their work and life and that their health status was generally poor despite regularly taking calcium therapy [40]. The Büttner et al. [41] survey found that patients with hypoparathyroidism after thyroid cancer surgery had significantly impaired QoL compared with patients without hypoparathyroidism. Our study is the first to evaluate the QoL of patients after parathyroid autotransplantation by the EORTC QLQ-C30 and THYCA QoL scales. The EORTC QLQ-C30 scale is the most widely used specific tool for assessing the QoL of cancer patients in European countries, and the THYCA QoL scale is currently the only thyroid cancer-specific QoL scale based on the C30 scale. We found that there was no significant difference in the quality of life between the two groups, especially in the symptom scale of hands and feet tingling. Indirectly, it was shown that the response to hypocalcemia symptoms was similar among populations with single inferior parathyroid autotransplantation and parathyroid preserved in situ.

There were several limitations to this study. First, this study was a single-center retrospective study, and selection bias may have been unavoidable. Second, the number of intraoperative identified parathyroid glands and the number of parathyroid glands retained in situ could not be effectively collected, which led to the difference in complications between the two groups of patients due to the inconsistent number of parathyroid glands. Finally, the absence of continuous Tg values in patients after surgery could not effectively evaluate the specific reasons for RAI treatment in patients in the two groups. Therefore, further larger and prospective studies are needed.

## Conclusion

In conclusion, single inferior parathyroid gland autotransplantation is associated with higher number of CLNs resected, without demonstrating differences in permanent hypoparathyroidism or structural central neck recurrence. Larger, randomized studies are warranted to better examine the potential benefits and risks of elective parathyroid autotransplantation.

## Data Availability

All data generated or analyzed during this study are included in this published article.

## Abbreviations

TT: Total thyroidectomy
CLND: Central lymph node dissection
PTC: Papillary thyroid carcinoma
QoL: Quality of life
CLN: Central lymph node
RAI: Radioactive iodine
LLN: Lateral lymph node
PTH: Parathyroid hormone
EORTC QLQ-C30: European Organization for Research and Treatment of Cancer Quality of Life Questionnaire-C30
THYCA-QoL: Thyroid Cancer-specific Quality of Life
LLND: Lateral lymph node dissection
CT: Computed tomography
IQR: Interquartile range
ATA: American Thyroid Association
Tg: Thyroglobulin
R: Retrospective
P: Prospective
BCLND: Bilateral central lymph node dissection
NA: Not acknowledge
SCM: Sternocleidomastoid muscle

## References

[1] Lim H, Devesa SS, Sosa JA, Check D, Kitahara CM (2017). Trends in thyroid cancer incidence and mortality in the United States, 1974–2013. JAMA.

[2] Liu YQ, Zhang SQ, Chen WQ (2012). Trend of incidence and mortality on thyroid cancer in China during 2003–2007. Zhonghua Liu Xing Bing Xue Za Zhi.

[3] Seib CD, Sosa JA (2019). Evolving understanding of the epidemiology of thyroid cancer. Endocrinol Metab Clin North Am.

[4] Harslof T, Rolighed L, Rejnmark L (2019). Huge variations in definition and reported incidence of postsurgical hypoparathyroidism: a systematic review. Endocrine.

[5] Xing Z, Qiu Y, Xia B (2021). Surgical strategy when identifying less than four parathyroid glands during total thyroidectomy: a retrospective cohort study. Gland Surg.

[6] Bliss RD, Gauger PG, Delbridge LW (2000). Surgeon's approach to the thyroid gland: surgical anatomy and the importance of technique. World J Surg.

[7] Gartland RM, Bloom JP, Parangi S (2019). A long, unnerving road: malpractice claims involving the surgical management of thyroid and parathyroid disease. World J Surg.

[8] Lorente-Poch L, Sancho JJ, Ruiz S, Sitges-Serra A (2015). Importance of in situ preservation of parathyroid glands during total thyroidectomy. Br J Surg.

[9] Sun R, Sheng J, Zhou Y (2021). Relationship between the extent of central node dissection and parathyroid function preservation in thyroid cancer surgery. Gland Surg.

[10] Kandil E, Noureldine SI, Abbas A, Tufano RP (2013). The impact of surgical volume on patient outcomes following thyroid surgery. Surgery..

[11] American Thyroid Association Surgery Working G, American Association of Endocrine S, American Academy of O-H, et al. Consensus statement on the terminology and classification of central neck dissection for thyroid cancer. Thyroid. 2009;19(11):1153–8. 10.1089/thy.2009.0159 19860578

[12] Orloff LA, Wiseman SM, Bernet VJ (2018). American Thyroid Association statement on postoperative hypoparathyroidism: diagnosis, prevention, and management in adults. Thyroid.

[13] Perrier ND, Brierley JD, Tuttle RM. Differentiated and anaplastic thyroid carcinoma: Major changes in the American Joint Committee on Cancer eighth edition cancer staging manual.[J] CA Cancer J Clin. 2018;68:55–63. 10.3322/caac.21439 PMC5766386 29092098

[14] Haugen BR, Alexander EK, Bible KC (2016). 2015 American Thyroid Association management guidelines for adult patients with thyroid nodules and differentiated thyroid cancer: the American Thyroid Association guidelines task force on thyroid nodules and differentiated thyroid cancer. Thyroid.

[15] Singer S, Jordan S, Locati LD (2017). The EORTC module for quality of life in patients with thyroid cancer: phase III. Endocr Relat Cancer.

[16] Giordano D, Valcavi R, Thompson GB (2012). Complications of central neck dissection in patients with papillary thyroid carcinoma: results of a study on 1087 patients and review of the literature. Thyroid.

[17] Su A, Wang B, Gong Y, Gong R, Li Z, Zhu J (2017). Risk factors of hypoparathyroidism following total thyroidectomy with central lymph node dissection. Medicine (Baltimore).

[18] Abboud B, Sleilaty G, Zeineddine S (2008). Is therapy with calcium and vitamin D and parathyroid autotransplantation useful in total thyroidectomy for preventing hypocalcemia?. Head Neck.

[19] Ahmed N, Aurangzeb M, Muslim M, Zarin M (2013). Routine parathyroid autotransplantation during total thyroidectomy: a procedure with predictable outcome. J Pak Med Assoc.

[20] Wei T, Li Z, Jin J (2014). Autotransplantation of inferior parathyroid glands during central neck dissection for papillary thyroid carcinoma: a retrospective cohort study. Int J Surg.

[21] Zedenius J, Wadstrom C, Delbridge L (1999). Routine autotransplantation of at least one parathyroid gland during total thyroidectomy may reduce permanent hypoparathyroidism to zero. Aust N Z J Surg.

[22] Lo CY, Lam KY (2001). Routine parathyroid autotransplantation during thyroidectomy. Surgery.

[23] Cavallaro G, Iorio O, Centanni M (2015). Parathyroid reimplantation in forearm subcutaneous tissue during thyroidectomy: a simple and effective way to avoid hypoparathyroidism. World J Surg.

[24] El-Sharaky MI, Kahalil MR, Sharaky O (2003). Assessment of parathyroid autotransplantation for preservation of parathyroid function after total thyroidectomy. Head Neck.

[25] Trupka A, Sienel W (2002). Autotransplantation of at least one parathyroid gland during thyroidectomy in benign thyroid disease minimizes the risk of permanent hypoparathyroidism. Zentralbl Chir.

[26] Fausto F, Palazzo MS, Sywak SB, Sidhu BH, Barraclough LW, Delbridge. Parathyroid Autotransplantation during Total Thyroidectomy—Does the Number of Glands Transplanted Affect Outcome? World J Surg. 2005;29(5):629-31. 10.1007/s00268-005-7729-9. 10.1007/s00268-005-7729-9 15827848

[27] Kihara M, Miyauchi A, Kontani K, Yamauchi A, Yokomise H (2005). Recovery of parathyroid function after total thyroidectomy: long-term follow-up study. ANZ J Surg.

[28] Wang B, Zhu C-R, Liu H, Wu J. The effectiveness of parathyroid gland autotransplantation in preserving parathyroid function during thyroid surgery for thyroid neoplasms: A meta-analysis. PLOS ONE. 2019;14(8):e0221173. 10.1371/journal.pone.0221173. 10.1371/journal.pone.0221173 PMC6693848 31412080

[29] Kirdak T, Dundar HZ, Uysal E, Ocakoglu G, Korun N (2017). Outcomes of parathyroid autotransplantation during total thyroidectomy: a comparison with age- and sex-matched controls. J Invest Surg.

[30] Lorente-Poch L, Sancho J, Munoz JL, Gallego-Otaegui L, Martinez-Ruiz C, Sitges-Serra A (2017). Failure of fragmented parathyroid gland autotransplantation to prevent permanent hypoparathyroidism after total thyroidectomy. Langenbecks Arch Surg.

[31] Selective Parathyroid Autotransplantation During Total Thyroidectomy for Papillary Thyroid Carcinoma: A Cohort Study Frontiers in Surgery. 2021. 10.3389/fsurg.2021.683041. 10.3389/fsurg.2021.683041 PMC8274712 34262932

[32] Su A, Gong Y, Wu W, Gong R, Li Z, Zhu J (2018). Effect of autotransplantation of a parathyroid gland on hypoparathyroidism after total thyroidectomy. Endocr Connect.

[33] Tartaglia F, Blasi S, Giuliani A (2016). Parathyroid autotransplantation during total thyroidectomy. Results of a retrospective study. Int J Surg.

[34] Robinson TJ, Thomas S, Dinan MA, Roman S, Sosa JA, Hyslop T (2016). How many lymph nodes are enough? Assessing the adequacy of lymph node yield for papillary thyroid cancer. J Clin Oncol.

[35] Yu ST, Ge JN, Sun BH (2021). Lymph node yield in the initial central neck dissection (CND) associated with the risk of recurrence in papillary thyroid cancer: a reoperative CND cohort study. Oral Oncol.

[36] Heaton CM, Chang JL, Orloff LA (2016). Prognostic implications of lymph node yield in central and lateral neck dissections for well-differentiated papillary thyroid carcinoma. Thyroid.

[37] Sung TY, Yoon JH, Song DE (2016). Prognostic value of the number of retrieved lymph nodes in pathological Nx or N0 classical papillary thyroid carcinoma. World J Surg.

[38] Piccardo A, Arecco F, Puntoni M, Foppiani L,Cabria M, Corvisieri S, Arlandini A, Altrinetti V, Bandelloni R, Orlandi F. Focus on High-Risk DTC Patients. Clin Nucl Med. 2013;38(1):18–24. 10.1097/RLU.0b013e318266d4d8. 10.1097/RLU.0b013e318266d4d8 23242039

[39] Polachek A, Hirsch D, Tzvetov G, et al. Prognostic value of post-thyroidectomy thyroglobulin levels in patients with differentiated thyroid cancer [J]. J Endocrinol Invest. 2011;34:855–60. 10.3275/7768 21646855

[40] Stevenson A, Mihai R (2018). Patients' views about parathyroid transplantation for post-thyroidectomy hypoparathyroidism. Langenbecks Arch Surg.

[41] Buttner M, Locati LD, Pinto M (2020). Quality of life in patients with hypoparathyroidism after treatment for thyroid cancer. J Clin Endocrinol Metab.

